# Distinct kinetic and mechanical properties govern mucin 16- and podocalyxin-mediated tumor cell adhesion to E- and L-selectin in shear flow

**DOI:** 10.18632/oncotarget.4704

**Published:** 2015-07-28

**Authors:** Daniel J. Shea, Denis Wirtz, Kathleen J. Stebe, Konstantinos Konstantopoulos

**Affiliations:** ^1^ Department of Chemical and Biomolecular Engineering, The Johns Hopkins University, Baltimore, Maryland; ^2^ Johns Hopkins Institute for NanoBioTechnology, The Johns Hopkins University, Baltimore, Maryland; ^3^ Johns Hopkins Physical Sciences-Oncology Center, The Johns Hopkins University, Baltimore, Maryland; ^4^ Department of Oncology, The Johns Hopkins University, Baltimore, Maryland; ^5^ Department of Chemical and Biomolecular Engineering, University of Pennsylvania

**Keywords:** Mucin16, PODXL, selectin, off-rate, pancreatic cancer

## Abstract

Selectin-mediated tumor cell tethering to host cells, such as vascular endothelial cells, is a critical step in the process of cancer metastasis. We recently identified sialofucosylated mucin16 (MUC16) and podocalyxin (PODXL) as the major functional E- and L-selectin ligands expressed on the surface of metastatic pancreatic cancer cells. While the biophysics of leukocyte binding to selectins has been well studied, little is known about the mechanics of selectin-mediated adhesion pertinent to cancer metastasis. We thus sought to evaluate the critical parameters of selectin-mediated pancreatic tumor cell tethering and rolling. Using force spectroscopy, we characterized the binding interactions of MUC16 and PODXL to E- and L-selectin at the single-molecule level. To further analyze the response of these molecular interactions under physiologically relevant regimes, we used a microfluidic assay in conjunction with a mathematical model to study the biophysics of selectin-ligand binding as a function of fluid shear stress. We demonstrate that both MUC16 and PODXL-E-selectin-mediated interactions are mechanically stronger than like L-selectin interactions at the single-molecule level, and display a higher binding frequency at all contact times. The single-molecule kinetic and micromechanical properties of selectin-ligand bonds, along with the number of receptor-ligand bonds needed to initiate tethering, regulate the average velocity of ligand-coated microspheres rolling on selectin-coated surfaces in shear flow. Understanding the biophysics of selectin-ligand bonds and their responses to physiologically relevant shear stresses is vital for developing diagnostic assays and/or preventing the metastatic spread of tumor cells by interfering with selectin-mediated adhesion.

## INTRODUCTION

E- and L-selectins play a vital role in cell-cell interactions pertinent to cancer metastasis. E-selectin (CD62E) is expressed on activated vascular endothelial cells and is thought to promote the tethering and rolling of malignant cells, acting as a critical step for their extravasation to the interstitial space and eventual seeding of new metastatic foci [1–3]. L-selectin (CD62L) is constitutively expressed on the surface of leukocytes, and promotes the recruitment of leukocytes to the metastasizing tumor microenvironment; deficiencies in L-selectin lessen metastatic spread in mice [4, 5]. Although E- and L-selectins share a similar structure, their binding kinetics and distribution differ considerably leading to distinct roles in cancer metastasis. At the lectin domain, selectins recognize sialofucosylated oligosaccharides such as sialyl-Lewis x (sLe^x^) and its isomer sialyl-Lewis a (sLe^a^) on interacting cells [1, 6–8]. Notably, sLe^x^ is absent from normal pancreas tissue [9, 10], but its expression is observed in pancreatic ductal adenocarcinoma and high-grade pancreatic intraepithelial neoplasia lesions [9]. Because the binding affinity of selectins for monocovalent sLe^x/a^ is low, it is vital to distinguish between structures that only interact with selectins *in vitro* and the *functional* selectin ligands [4] that interact with selectins selectively and with high affinity *in vivo* and whose depletion suppresses selectin-dependent binding. We recently identified sialofucosylated mucin 16 (MUC16) and podocalyxin (PODXL) as the major functional E- and L-selectin ligands expressed on the surface of metastatic pancreatic cancer cells [11, 12].

PODXL is a transmembrane glycoprotein that belongs to the CD34 family of proteins. Clinically, PODXL expression is associated with highly aggressive tumors and poor prognosis in several cancers [13] including invasive colorectal cancer [14]; it has also been identified as a diagnostic marker for pancreatic ductal adenocarcinomas originating in gastrointestinal and bile ducts [15]. MUC16 is a heavily glycosylated transmembrane protein, which is expressed in epithelial ovarian cancer [16, 17] and metastatic pancreatic cancer cells but not in normal pancreatic cells [11, 18]. Although both MUC16 and PODXL serve as functional E-/L-selectin ligands [11, 12], little is known about the mechanical properties of these interactions.

To determine the mechanical properties of the PODXL/MUC16-E-/L-selectin interactions, we utilized single-molecule force spectroscopy and microfluidic adhesion assays coupled with a mathematical model. Using force spectroscopy, we determined the kinetic ($k_{\text{off}}^{{}^{\circ}}$) and micromechanical (*x_β_* and tensile strength) properties of receptor-ligand interactions at the single-molecule level. By combining these measurements with microfluidic adhesion assays and mathematical modeling, we determined that the $k_{\text{off}}^{{}^{\circ}}$ and tensile strength of these selectin-ligand bonds, along with the number of bonds needed to mediate tethering, regulate the velocity of ligand-coated microspheres rolling on selectin-coated surfaces in shear flow. This integrated approach contributes to the understanding of how two major pancreatic cancer surface glycoproteins bind to E-/L-selectins in the presence of hydrodynamic shear, which can lead to the improved diagnosis and prevention of the metastatic spread of pancreatic cells.

## RESULTS

### Comparison of the kinetic and micromechanical properties of PODXL/MUC16-E-/L-selectin via single-molecule force spectroscopy

**Figure 1:**
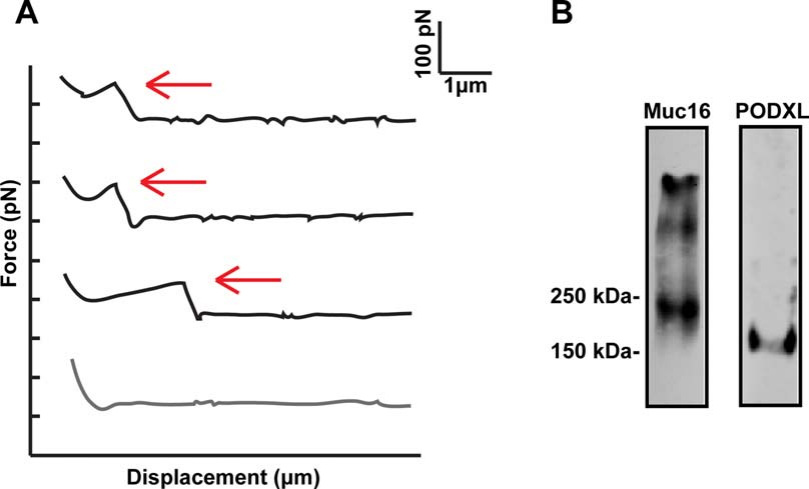
Single molecule force spectroscopy Force Displacement traces and western blots image **A.** Typical force-displacement traces acquired from force spectroscopy experiments where an E-/L-selectin fuctionalized cantilever was brought in contact hundreds of times with either immunopurified PODXL or MUC16 incorporated in a lipid vesicle deposited on a PEI-cushioned glass slide. Red arrows indicate rupture events with the loading rate estimated based on the slope of the linear ramping in force immediately before bond rupture. **B.** MUC16 and PODXL were immunopurified from SW1990 cells and were subjected to SDS-PAGE under reducing conditions followed by immunoblotting with an anti-PODXL mAb (3D3) or anti-MUC16 mAb (M11).

Single-molecule force spectroscopy has been utilized to determine the kinetic properties of selectin-ligand bonds in both purified protein-protein and protein-cell systems [19–21]. Incorporation of the purified protein (e.g., MUC16 or PODXL) into a lipid bilayer allows for its physiologically relevant orientation and provides a consistent binding interface, while eliminating the potential contribution of other selectin ligands present on the tumor cell surface [19, 20, 22]. Here, we utilize single-molecule force spectroscopy to evaluate the binding strength of MUC16 and PODXL interactions with both E- and L-selectin. In this assay, E- or L- selectin-coated cantilevers were brought in contact with immunopurified MUC16 or PODXL inserted in a lipid bilayer for a constant dwell time and retracted at designated retraction speeds. The bond rupture force (tensile strength) and loading rates of single binding events were measured over a range of retraction velocities (Figure 1A) [20, 22]. Dilute E-/L-selectin and ligand concentrations on the cantilever tips and lipid bilayer, respectively, were selected to ensure single bond formation [20, 22]. To validate the measurements obtained using immunopurified MUC16 and PODXL (Figure 1B), we also tested E-/L-selectin binding to MUC16-expressing (PODXL-knockdown) and PODXL-expressing (MUC16-knockdown) SW1990 pancreatic cancer cells [11, 12].

**Figure 2:**
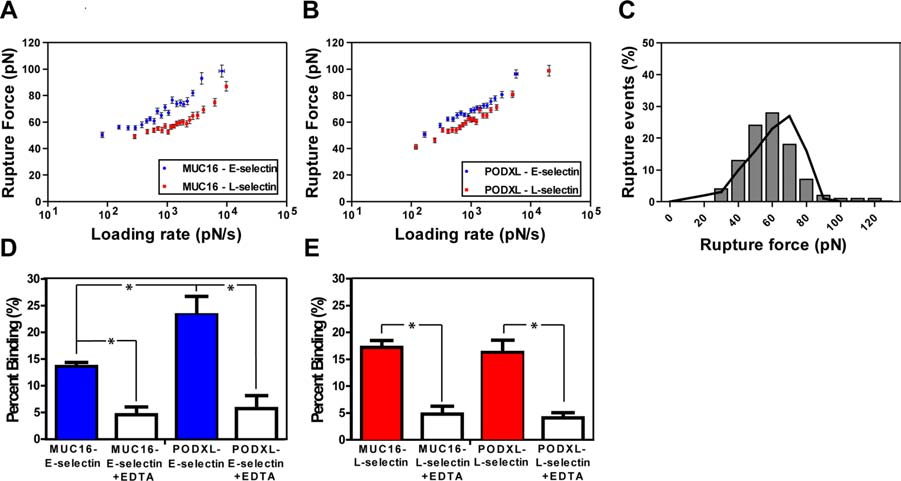
Micromechanical properties of immunopurified MUC16/PODXL binding to E/L-selectin using single molecule force spectroscopy Rupture force as a function of loading rate for immunopurified **A.** MUC16-E/L-selectin and **B.** PODXL-E/L-selectin interaction. Data represent the mean ± S.E.M. of 3–4 experiments run for each binding pair. Least-squares fitting of the Bell model were used to determine kinetic parameters. **C.** Rupture force distribution was obtained both experimentally (bars) and computationally through Monte-Carlo simulations (line) based on Bell model kinetic parameters for immunopurified PODXL-E-selectin binding. **D-E.** Frequency of binding between immunopurified MUC16 and PODXL with E-selectin and L-selectin in the absence and presence of the divalent cation chelator EDTA. Tips coated with 10 μg/ml E-/L-selectin were brought in contact with either MUC16 or PODXL incorporated lipid bilayers. The frequency of binding was measured with and without the presence of 10 mM EDTA. Data represent the mean ± S.E.M. of 3–4 independent experiments. *P < 0.05.

**Figure 3:**
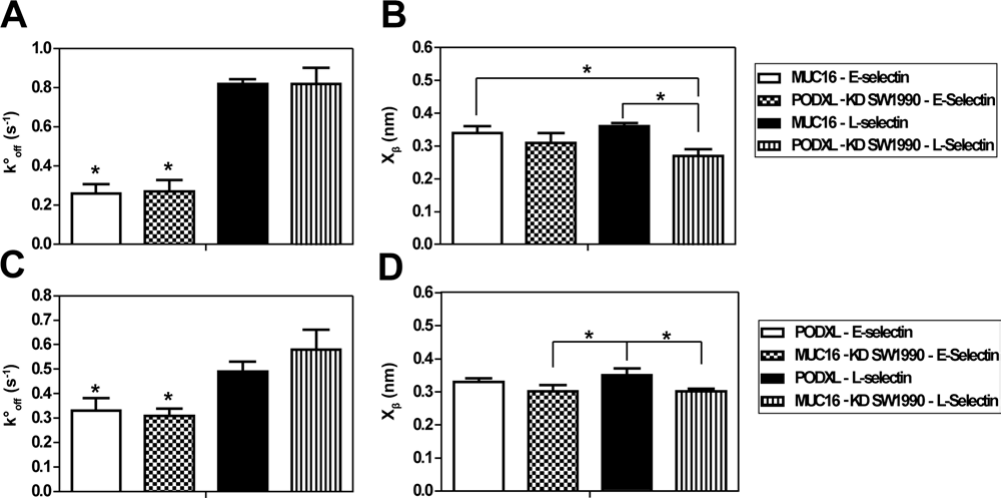
Bell Model kinetic properties of protein-protein and cell-protein interactions **A, C.** Unstressed off-rate (s^−1^) and **B, D.** reactive compliance (nm) for MUC16 and PODXL interactions with E/L-selectin using a range of retraction velocities (5–25 μm/s). Data represent mean ± S.D. of 3–5 independent experiments which each had at least 1, 250 approach/retract cycles. Cantilever tips coated with E/L-selectin were brought in contact with immunopurified MUC16 or PODXL incorporated lipid bilayers or MUC16-expressing (PODXL-KD) or PODXL-expressing (MUC16-KD) SW1990 cells. *P < 0.05 with respect to both MUC16-L-selectin and PODXL-KD SW1990-L-selectin in (A). *P < 0.05 with respect to both PODXL-L-selectin and MUC16-KD SW1990-L-selectin interactions in (C). *P < 0.05 with respect to the indicated interactions (B, D).

For all interactions tested, the rupture force varied linearly with the natural log of the bond-loading rate for nearly the entire loading rate regime (Figure 2A, 2B). The rupture force-loading plots were similar for both immunopurified ligand-selectin pairs (Figure 2A, 2B) and their respective knockdown cell-selectin counter parts (Supplementary Figure S1). Mean rupture force and rupture force distribution were similar for both protein-protein and cell-protein systems (e.g., 71 pN for MUC16-E-selectin and 74 pN for MUC16-expressing (PODXL-knockdown) SW1990-E-selectin at 1000 pN/s). Using the least-squares fit to the plot of mean rupture force versus the natural log of the loading rate the Bell models parameters, unstressed off-rate $k_{\text{off}}^{{}^{\circ}}$ (s^−1^) and reactive compliance *x_β_*(nm) were determined for all E-/L-selectin interactions. Both protein-protein and cell-protein systems displayed similar kinetic constants for each pair of interactions (Figure 3). Specifically, the $k_{\text{off}}^{{}^{\circ}}$ was nearly identical for all respective pairs, while *x_β_* was moderately lower for the cell-L-selectin system compared to the immunopurified MUC16/PODXL-L-selectin system (Figure 3), likely due to the minor differences in protein glycosylation [23, 24]. This is in line with prior observations showing that knockdown of a sialofucosylated protein (e.g., CD44v) increases the extent of glycosylation (HECA-452 reactivity) in other glycoproteins (e.g., PODXL) [23, 24]. Overall, given the similarities in the mechanical properties of both systems, we conclude that immunopurified MUC16 and PODXL incorporated bilayers represent a reliable system for measuring the interactions between these ligands and E-/L-selectin on the single-molecule level.

The binding frequency for immunopurified MUC16 with E-/L-selectin was 13.6% and 17.3%, respectively (Figure 2D, 2E). While the binding frequency of PODXL-L-selectin interaction was in the same range at 16.3%, it was notably higher at 23.3% for the PODXL-E-selectin pair (Figure 2D, E), suggesting a higher on-rate. The addition of EDTA (10 mM) suppressed the binding of all interacting pairs down to basal level (∼5%), which is in accordance with the Ca^2+^ dependence of E- and L-selectin-dependent binding [25]. These findings, in conjunction with the minimal binding found with a selectin-blank bilayer interaction (data not shown), help verify the specificity of the selectin-ligand interactions studied here.

The MUC16-E-selectin bond displayed a higher rupture force (tensile strength) at all loading rates when compared to the MUC16–L-selectin bond (71 pN to 56 pN, respectively, at a loading rate of 1000 pN/s) (Figure 2A). The $k_{\text{off}}^{{}^{\circ}}$ was found to be markedly lower for the MUC16-E-selectin bond (0.26 s^−1^) than for the MUC16-L-selectin bond (0.82 s^−1^), whereas no significant differences were observed for *x_β_* (Table 1). Similarly, the PODXL-E-selectin bond displayed a higher rupture force at all loading rates than the PODXL-L-selectin bond (e.g., 69 pN to 62 pN, respectively, at a loading rate of 1000 pN/s) (Figure 2B). The $k_{\text{off}}^{{}^{\circ}}$ was lower for the PODXL-E-selectin bond (0.33 s^−1^) when compared to the PODXL-L-selectin dissociation (0.49 s^−1^), while no significant difference was found for *x_β_*. Bell model parameters for all binding pairs were validated using Monte Carlo simulations (Figure 2C). Taken together, both MUC16 and PODXL-E-selectin bonds were determined to be mechanically stronger than the like ligand-L-selectin bonds, as evident by the higher tensile strength, lower bond off-rate and thus lower susceptibility to rupture. Although MUC16- and PODXL-E-selectin bonds have similar kinetic properties and tensile strengths, the PODXL-L-selectin bond is mechanically stronger than the MUC16-L-selectin bond.

**Table 1:** Bell model parameters for PODXL and MUC16-E/L-selectin interactions

Interaction	$k_{\text{off}}^{{}^{\circ}}\;$(*s*^−1^)	*x_β_*(nm)
MUC16-E-selectin	0.26 ± 0.08	0.34 ± 0.02
MUC16-L-selectin	0.82 ± 0.04	0.36 ± 0.01
PODXL-E-selectin	0.33 ± 0.05	0.33 ± 0.01
PODXL-L-selectin	0.49 ± 0.04	0.35 ± 0.02

Bell model parameters were determined using a least-squares fit to the plot of linear region of rupture force against the logarithm of loading rate. Data are shown as the mean ± S.D.

### Single-molecule kinetic and micromechanical properties along with contact time and site density dictate MUC16 and PODXL binding to E-/L-selectins under flow

To study the biophysics of MUC16/PODXL-selectin binding under flow, we quantified the extent of MUC16- and PODXL-coated microspheres binding to selectins as a function of contact time and selectin site density. Receptor-ligand contact time is regulated by the shear stress and length of the selectin-coated surface in the direction of flow. Using photolithography, E- and L-selectin-coated patches with lengths varying from 10–160 μm and a width of 10 μm were micropatterned onto a glass slide [26]. The patterns were spaced 100 μm apart in the direction of flow and selectin-free regions were coated with inert bovine serum albumin (BSA). Over the course of the 3-min experiment, polystyrene microspheres (2 × 10^6^/ml) coated with either MUC16 or PODXL at equivalent site densities (∼20 sites/μm^2^, Supplementary Figure S2) were perfused over the micropatches at prescribed wall shear stresses. Of note, this site density is in the same range of site densities reported in literature [27–29]. The number of ligand-coated microspheres interacting on each patch for each experiment was recorded (N_b_). The fraction of microspheres binding to each patch (N_b_/N_T_) was determined by dividing the interacting microspheres (N_b_) by the total number of microspheres to flow over the patch (N_T_).

**Figure 4:**
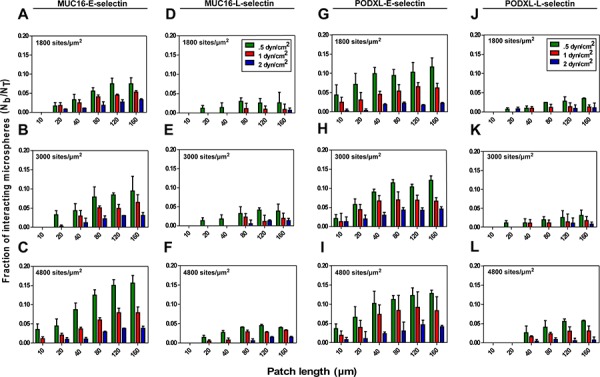
Fraction of interacting microspheres with E/L-selectin coated patches at designated shear stresses Microspheres (2 × 10^6^/ml) coated with either MUC16 or PODXL at equivalent site densities flowed at prescribed shear stresses over glass surfaces containing patches coated with three site densities of either E- or L-selectin. The number of MUC16 **A-F.** or PODXL **G-L.** coated microspheres interacting with each patch was counted and normalized based on the microsphere flux over each patch. Data represent the fraction of interacting microspheres (N_b_/N_T_) mean ± S.E.M. of at least three experiments.

Longer selectin patch lengths increased the fraction of interacting microspheres as ligand-selectin contact time increases with increasing patch lengths (Figure 4). Along these lines, we also observed a consistent increase in the fraction of interacting microspheres with decreasing shear stresses and thus correspondingly increasing contact times for all tested interactions (e.g., 4.6%, 6.6% and 12.1% capture efficiency for PODXL-E-selectin interaction using 160 μm long patches coated with E-selectin (3000 sites/μm^2^) at 2, 1 and 0.5 dyn/cm^2^, respectively) (Figure 4H). This monotonic relationship between capture efficiency and shear stress indicates that the shear threshold effect, which is observed in leukocyte tethering [30], does not occur for MUC16- or PODXL-selectin interactions in the shear stress regime studied here.

To investigate the effect of selectin density on capture efficiency, the slides were incubated with three concentrations of E- or L-selectin. Using a modified europium assay [26], we determined the surface site densities of E- and L-selectin plated at concentrations of 5, 10 and 20 μg/ml to be approximately 1800, 3000, and 4800 sites/μm^2^, respectively (Supplementary Figure S3). These site densities are in a range similar to those reported in literature [26, 31, 32]. Decreasing selectin densities decrease binding for all selectin-ligand pairs (e.g., 7.9%, 6.5% and 5.5% for MUC16-E-selectin interaction at 1 dyn/cm^2^ using 160 μm long patches) (Figure 4A–4C). Ligand-L-selectin binding followed a similar trend; however, the fraction of interacting microspheres was considerably lower than that for ligand-E-selectin interactions (e.g., 3.3%, 1.9% and 0.9% for MUC16-L-selectin interaction at 1 dyn/cm^2^ using 160 μm patches) (Figure 4D–4F). Taken together, the higher tensile strength and lower $k_{\text{off}}^{{}^{\circ}}$ measured by single-molecule force spectroscopy predict the enhanced capacity of MUC16 and PODXL to bind to E-selectin over L-selectin.

The critical patch length (CPL), defined as the minimum patch length to achieve at least 2% binding, increases with increasing shear stress and decreases with increasing selectin density for both the MUC16 and PODXL-E-selectin interactions (Table 2). In line with the force spectroscopy data, the critical patch length was longer for ligand-L-selectin interactions when compared to E-selectin interactions at all shear stresses and selectin site densities studied indicating that the weaker L-selectin-mediated binding requires lower shear stresses and higher selectin site densities to achieve similar binding.

**Table 2:** Critical patch length of PODXL and MUC16–E/L-selectin interactions

	MUC16-E-selectin and (L-selectin)			PODXL-E-selectin and (L-selectin)		
	Selectin density (sites/μm^2^)					
Shear stress (dyn cm^−2^)	1800	3000	4800	1800	3000	4800
2	120 (NB)	80 (NB)	80 (NB)	160 (NB)	40 (NB)	40 (NB)
1	40 (NB)	40 (NB)	20 (80)	40 (NB)	20 (NB)	20 (80)
0.5	40 (80)	20 (80)	10 (40)	10 (80)	10 (80)	10 (40)

NB indicates no binding. The critical patch length of selectin is defined as the minimum patch length required for MUC16 or PODXL coated microshperes to initiate 2% binding at a given shear stress and selectin site density. The numbers inside the parentheses indicate the critical patch length for L-selectin.

### Multiple bonds are needed for tethering to selectins under flow

**Figure 5:**
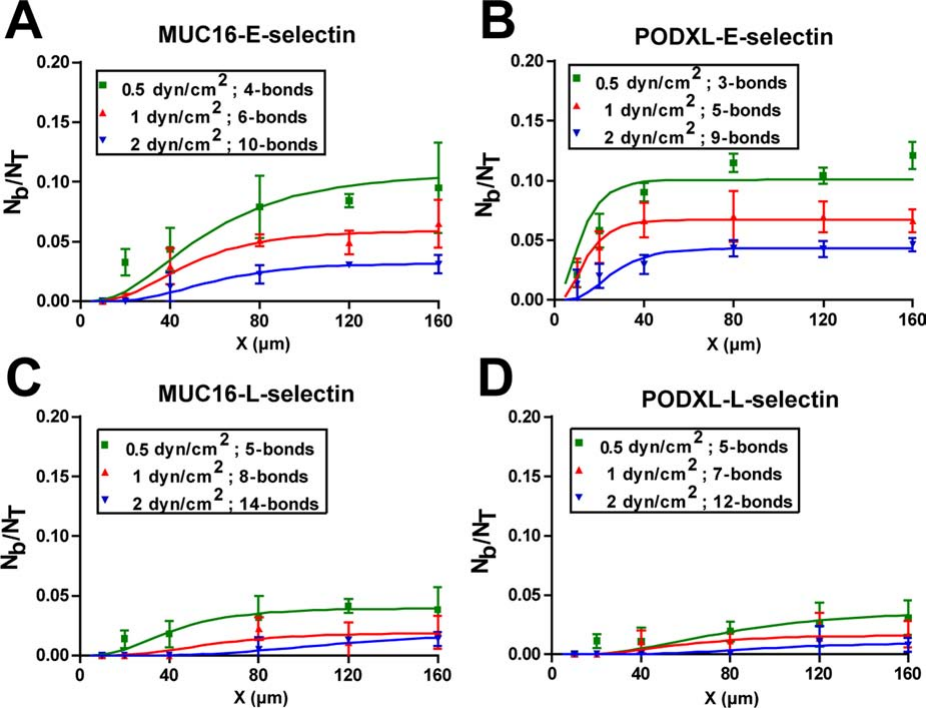
Fitting of the multi-bond model to the experimental data Immunopurified MUC16 **A, C.** or PODXL **B, D.** coated microspheres flowed over glass surfaces patterned with 3000 sites/μm^2^ E-selectin (A, B) or L-selectin (C, D) coated patches of varying lengths. The multi-bond model was fitted to the experimental data (points) and optimized for both A_c_M_r_M_l_K_on_ and n-bonds, with the solid lines representing the model prediction.

As shear stress increases, multiple selectin-ligand bonds are often needed to mediate cell tethering to a surface [26, 33]. To determine the number of MUC16/PODXL-selectin bonds required to mediate microsphere tethering in shear flow, we fitted the experimental binding data with an analytical multiple bond model [26]. This model estimates not only the minimum number of bonds needed for tethering but also the lumped binding affinity (A_c_M_r_M_l_K_on_) by simultaneously optimizing the two parameters (Supplementary Figure S4). The model successfully captured the binding profile for all selectin-ligand pairs examined (Figure 5). For all cases, the bond number increased with increasing shear stress. However, the minimum bond number varied for the each interaction based on their binding profiles.

According to the model, the minimum bond number for MUC16-coated microspheres tethering to E-selectin ranges from 4 to 10 when the shear stress increases from 0.5 to 2 dyn/cm^2^ (Figure 5A). In comparison, the PODXL-E-selectin interaction requires one less bond to mediate binding (3–9 bonds) in shear flow (0.5–2 dyn/cm^2^) (Figure 5B). When fewer bonds are required for tethering, such receptor-ligand interactions can be initiated at lower contact times, and thus shorter patches, as is the case for PODXL-E-selectin relative to MUC16-E-selectin (Figure 5A, 5B). For both MUC16/PODXL-E-selectin interactions, bonds increase in a shear dependent manner (Figure 5A, 5B). A similar shear-dependent trend was noted for the MUC16/PODXL-L-selectin interaction (Figure 5C, 5D). While both MUC16/PODXL-L-selectin interactions are mediated by 5 bonds at 0.5 dyn/cm^2^, fewer bonds are required for PODXL-L-selectin than MUC16-L-selectin binding at higher shear stresses (Figure 5C, 5D).

The lumped affinity term A_c_M_r_M_l_K_on_ considers the interacting contact area (A_c_), the receptor site density (M_r_), the ligand site density (M_l_) and the binding affinity (K_on_) calculated for each interaction. Given the equivalent values of M_r_ and M_l_ for all samples, the lumped binding affinity is a direct measure of 2-D binding affinity (A_c_K_on_). The model predicts that the PODXL tethering to E-selectin has a ∼2 fold higher A_c_M_r_M_l_K_on_ than MUC16-E-selectin at shear stresses ranging from 0.5–2 dyn/cm^2^ (Supplementary Table S1). This result is in line with the increased binding frequency measured for the PODXL-E-selectin pair by single-molecule force spectroscopy (Figure 2D). In contrast, the lumped affinity is modestly higher for the MUC16-L-selectin than the PODXL-L-selectin pair (Supplementary Table S1).

### Single-molecule kinetic and micromechanical properties along with bond number regulate MUC16 and PODXL-dependent rolling on selectins in shear flow

**Figure 6:**
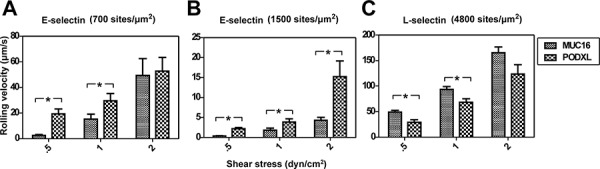
Average rolling velocities of MUC16- and PODXL-coated microspheres on E/L-selectin Average rolling velocities (μm/s) of microspheres (2 × 10^6^/ml) coated with either MUC16 or PODXL at equivalent site densities on immobilized E-selectin **A, B.** or L-selectin **C.** Data were recorded at prescribed wall shear stresses and represent the mean ± S.E.M. of at least 3 independent experiments. *P < 0.05.

Microspheres (2 × 10^6^/ml) coated with MUC16 or PODXL at equivalent site densities (∼20 sites/μm^2^) were perfused over glass slides coated with either E-selectin (700 and 1500 sites/μm^2^) or L-selectin (4800 sites/μm^2^). These selectin site densities were chosen as the perfused microspheres displayed clear rolling behavior. Slower rolling of ligand-coated microspheres was observed on E-selectin as compared to L-selectin substrates (Figure 6). As expected, microspheres rolled more slowly on surfaces coated with higher E-selectin density (1500 versus 700 sites/μm^2^) (Figure 6A, B), as this increases the number of receptors available for binding. On E-selectin substrates, MUC16-coated microspheres rolled significantly slower than PODXL-coated microspheres at the higher E-selectin density and at all shear stress tested (Figure 6B) even though the single-molecule kinetic and micromechanical properties are similar for MUC16 and PODXL interactions with E-selectin. This difference in rolling velocity is attributed to the higher number of MUC16 versus PODXL bonds with E-selectin needed to support tethering (Figure 5A, B). As such, the same loading rate is distributed among more MUC16-E-selectin than PODXL-E-selectin bonds of similar tensile strength for individual bonds, thereby supporting slower MUC16-dependent rolling at each prescribed shear stress level (Figure 6B). On L-selectin surfaces, both ligands displayed a rather transient rolling behavior, with PODXL-coated microspheres rolling significantly slower than MUC16-coated microspheres at lower (0.5 and 1 dyn/cm^2^) but not at the higher shear stress of 2 dyn/cm^2^ (Figure 6C). In the low shear regime (0.5 dyn/cm^2^), both MUC16- and PODXL-coated microspheres tether to L-selectin with an identical bond number (Figure 5C, D). Thus, the slower rolling velocity mediated by PODXL is attributed to lower $k_{\text{off}}^{{}^{\circ}}$ and higher tensile strength of PODXL-L-selectin interaction (Supplementary Table S2). At the higher shear stress level of 2 dyn/cm^2^, the difference in rolling velocity is no longer statistically significant because the differences in the kinetic and micromechanical properties of single bonds between PODXL versus MUC16 with E-selectin are masked by the higher number of MUC16-L-selectin bonds (Figure 5C, 5D) required to support tethering.

## DISCUSSION

The adhesive interactions between selectins on host cells and their respective ligands expressed on tumor cells are known to facilitate tumor cell arrest in the microvasculature and promote cancer metastasis [1, 3]. While the biophysics of leukocyte binding to selectins has been well studied [34–36], little is known about the mechanics of selectin-mediated tumor cell adhesion. As circulating tumor cells contact the blood vessel wall, selectin-mediated interactions support rolling and facilitate subsequent firm adhesion, making them a vital part of the metastatic cascade. Cell adhesion relies on the balance between the adhesive forces of receptor-ligand pairs and disruptive hydrodynamic forces [37]. We herein employed force spectroscopy and a microfluidic assay in conjunction with a mathematical model to delineate the contributions of the single-molecule kinetic and micromechanical properties of selectin-ligand bonds as well as the number of selectin-ligand bonds to pancreatic cancer cell tethering and rolling in shear flow. A quantitative analysis of receptor-ligand binding kinetics of *functional* selectin ligands expressed by metastatic tumor cells, such as MUC16 and PODXL, in the presence of shear flow will further the understanding of the metastatic process.

P-selectin glycoprotein-1 (PSGL-1) expressed by leukocytes is a known ligand of P-, E- and L-selectin. PSGL-1-selectin interactions have been extensively studied using diverse biophysical assays [21, 30, 34, 35, 38–41]. Although the unstressed off-rate, $k_{\text{off}}^{{}^{\circ}}$, is similar for PSGL-1, MUC16 and PODXL binding to E-selectin, the PSGL1-E-selectin bond is markedly stronger than MUC16-, PODXL-E-selectin bonds, as evidenced by its lower reactive compliance *x_β_*, and higher tensile strength (Supplementary Table S2) [42]. Similarly, the PSGL-1-L-selectin bond displays a lower reactive compliance and higher rupture force than MUC16-, PODXL-L-selectin bonds (Supplementary Table S2). As expected, the single-molecule bond of E- or L-selectin with monovalent sLe^x^ is markedly weaker than all aforementioned respective ligand-selectin pairs (Supplementary Table S2) [43].

Cell binding to a substrate occurs when an individual bond or a cluster of bonds are formed under flow that are capable of withstanding the dispersive hydrodynamic forces, thereby tethering the cell to the surface. Because the single bonds between MUC16 or PODXL with E-selectin are stronger than the respective ones with L-selectin (Table 1), fewer bonds are required to mediate microsphere tethering to E-selectin than L-selectin in shear flow. As such, the binding frequency of MUC16- or PODXL-coated microspheres is higher for E-selectin than L-selectin at equivalent site densities and shear stresses. Along these lines, the critical patch length is lower for E-selectin than L-selectin. It is noteworthy that while MUC16 and PODXL bonds with E-selectin have similar tensile strengths, a shorter critical patch length is required for the PODXL-E-selectin interaction. This is due to the lower number of bonds needed to tether PODXL-versus MUC16-coated microspheres to an E-selectin-coated surface (Figure 5A, B) coupled with its higher affinity (Supplementary Table 1). Also, the lower number of bonds required to mediate tethering of PSGL-1-expressing cells to L-selectin coupled with its higher tensile strength relative to MUC16- or PODXL-L-selectin bond can help explain the markedly higher binding frequency of PSGL-1-expressing cells to L-selectin micropatches [26].

A plateau in the extent of PODXL- and MUC16- dependent tethering to E-selectin is observed for patch lengths longer than 40 and 80 μm, respectively, where a further increase in patch length no longer increases binding. The plateau initiation point was found to directly relate to the critical patch length and the apparent multiple bond off-rate in the presence of force (*k_off,n_*), which considers the unstressed off-rate and reactive compliance of the single bond ($k_{\text{off}}^{{}^{\circ}}$ and $x_{\beta }$) as well as the number of bonds formed (*n*), and the hydrodynamic force on the microsphere (*f*) [33]. As shown in Eqn. 1, we assumed that at the initiation of microsphere tethering, the hydrodynamic force is distributed evenly among every bond, and when *n*-bonds break serially they do so in a stepwise fashion such that the force is redistributed among the remaining bond(s).

$$k_{\text{off},n}=\;\frac{1}{\sum_{i=1}^{n}\frac{1}{\;k_{\text{off},i}}}=\;\frac{1}{\sum_{i=1}^{n}\frac{1}{\;k_{\text{off}}^{o}\exp\left(\frac{x_{\beta }f}{ik_{B}T}\right)}}$$ (1)

The microsphere is assumed to dissociate when all bonds have ruptured, and interaction time is calculated by summing the total time needed to dissociate all bonds. Here, higher values for the apparent *k_off, n_* can result from either decreased bond strength or the formation of fewer bonds. In the case of E-selectin binding, single-bond tensile strength is similar for both PODXL and MUC16, and the faster plateauing of the PODXL-E-selectin interaction results from the lower bond number required for tethering.

Several studies have reported slower rolling on E-selectin versus L-selectin due to the lower unstressed off-rate $k_{\text{off}}^{{}^{\circ}}$ associated with E-selectin binding [39, 44]. We also see this trend; however, it appears that the number of bonds needed for adhesion in shear flow also plays an important role in regulating the rolling velocities of MUC16- and PODXL-coated microspheres on E- and L-selectin. Although the $k_{\text{off}}^{{}^{\circ}}$ of single bonds between MUC16 and PODXL with E-selectin are similar, we observed significant differences in their respective rolling velocities. As determined by fitting the multi-bond model to the experimental data, an additional bond is needed to initiate MUC16-E-selectin compared to PODXL-E-selectin binding (Figure 5A, 5B). As such, the apparent *k_off, n_* is lower for MUC16-E-selectin at all shear stress levels. If we consider microsphere rolling to consist of two steps: (i) the microsphere moving either at the hydrodynamic velocity (V = U_d_) or (ii) it is bound (with V = 0), this result makes sense. A larger number of bonds must be broken to release the microsphere from the surface; the presence of more bonds allows the microsphere to spend more time in the bound state, thereby slowing the rolling velocity provided that the on-rate is significantly fast. This behavior is also noted for L-selectin binding where at 0.5 dyn/cm^2^ both MUC16- and PODXL-coated microspheres require 5 bonds for binding, and rolling is $k_{\text{off}}^{{}^{\circ}}$ dependent. At the shear stress level of 2 dyn/cm^2^, the increased number of bonds needed for MUC16-L-selectin tethering acts as a natural break, slowing its rolling to similar levels to those of PODXL-L-selectin indicating that bond clusters may play a critical role in cell rolling stability [45, 46]. Differences in rolling velocities between E- and L-selectin pairs likely also result from differences in bond tensile strength that has been reported to influence rolling [21, 42]. Therefore, the interplay of the tensile strength, $k_{\text{off}}^{{}^{\circ}},$ and bond number regulates the rolling velocities of MUC16- and PODXL-coated microspheres on E- and L-selectins. In addition to these parameters, cell deformation affects rolling in shear flow [47].

MUC16 and PODXL have been identified as *functional* E- and L-selectins ligands that are overexpressed in metastatic pancreatic cancer cells [11, 12]. In this study, we utilized the combination of single-molecule bond characterization and microfluidic assays coupled with a mathematical model to characterize ligand-dependent tethering and rolling in physiologically relevant flow conditions. We found that the single-molecule kinetic and micromechanical properties and the number of bonds needed for tethering predict the binding interactions and rolling of MUC16- and PODXL-coated microspheres on E-/L-selectin coated surfaces. This integrated approach contributes to a better understanding of how MUC16 and PODXL bind to E-/L-selectins in the presence of hydrodynamic shear, which can lead to improved diagnostic assays and to the prevention of the metastatic spread of pancreatic tumor cells.

## MATERIALS AND METHODS

### Reagents and monoclonal antibodies

Anti-human PODXL monoclonal antibody (3D3) was from Santa Cruz Biotechnology (Santa Cruz, CA) while the anti-human MUC16 monoclonal antibody (M11) was from Dako (Carpinteria, CA). PEI was from Polysciences (Warrington, PA). DMPC was from Avanti Polar Lipids (Alabastar, AL).

### Cell culture, whole cell lysis and immunoprecipitation

Human pancreatic adenocarcinoma SW1990 cells were obtained from the American Type Culture Collection (Manassas, VA). SW1990-PODXL-KD and SW1990-MUC16-KD cells were generated as previously described [11, 12]. All SW1990 cells were cultured in DMEM with 10% FBS with 700 μg/ml G418 and 0.5 μg/ml puromycin added to the PODXL-KD and Mucin16-KD media, respectively (Life Technologies, Carlsbad, CA). SW1990 whole-cell lysate was prepared through treatment with 2% Nonidet P-40 followed by centrifugation [11, 12]. PODXL and MUC16 were immunopurified using anti-PODXL mAb (3D3) or anti-MUC16 mAb (M11), respectively, using protein G agarose beads (Invitrogen) [23–25, 48].

### Lipid bilayer preparation

To prepare the solutions used for the lipid bilayer, 8 mg of DMPC (1, 2-dimyristoyl-sn-glycero-3-phosphocholine) was added to 8 mL of lipid buffer B (Tris-HCl, 50 mM NaCl, 1 mM CaCl_2_, 0.1% (w/v) Triton X-100) [19, 20, 22, 49]. 130 μL of either MUC16 or PODXL (concentration 80 μg/ml) was added to 370 μl of the lipid solution and resulting solution was incubated at 37°C for 2 h. Following incubation, the solution was transferred to 10 kDa MWCO dialysis cassette and dialyzed against 1 L of lipid buffer A (20 mM Tris-HCl, 50 mM NaCl, 1 mM CaCl2). Buffer A was changed three times every 12 h. After 48 h the dialyzed solution was stored at 4°C under nitrogen. Glass slides were plasma cleaned for 5 min and then incubated with 100 ppm PEI in 0.5 mM KNO_3_ for 20 min. Following cleaning with DI water and air, slides were placed in a vacuum desiccator for at least 2 h before use. 4 μL protein-lipid solution was added to the slide for 2 h and the droplet was maintained using a moist towel. Slides were washed 3X using Hank's Balanced Salt Solution (HBSS) before being submerged in HBSS for use in experiments. In select experiments, 10 mM EDTA was added to the HBSS and slides were submerged in the EDTA/HBSS solution.

### Cantilever functionalization

Molecular force probe cantilevers from Bruker nano (Camarillo, CA) were silanized with 2% (v/v) 3-amino-propyltriethoxysilane in acetone [19, 42]. Cantilevers were incubated for 1 h in 30 μg/ml anti-human IgG-Fc mAb (Abcam, Cambridge, MA) in D-PBS containing a 50-fold molar excess of the bis(sulfosuccinimidyl) croslinker (BS^3^; Peirce, Rockford, IL) before quenching with Tris buffer. Cantilevers were then incubated in either 10 μg/ml E-selectin Fc chimera or 10 μg/ml L-selectin Fc chimera (R&D systems, Minneapolis, MN) in D-PBS for 2 h at room temperature and blocked with 1% BSA in D-PBS to limit nonspecific interactions. The concentrations of selectins were optimized to ensure for a low proportion of binding events in force spectroscopy experiments (∼20 per 100 contacts).

### Single molecule force spectroscopy and data acquisition

Experiments were conducted using a MFP (Asylum Research, Santa Barbara, CA). A triangular cantilever tip with spring constant of 10 pN/nm was calibrated using thermal noise amplitude. The deflection was measured by laser reflection on a split photodetector. The petri-dish containing the lipid bilayer or SW1990 cells was positioned directly under the cantilever and adjusted so that each approach generated a slight depression force (∼5 nN) on the sample before reproach. The dwell time was set to 20 msec and the reproach velocity was varied from 5–25 μm/sec. Rupture forces and loading rates were determined from force-distance traces using Igor Pro 4.09 software (Wavemetrics, Lake Oswego, OR). For each receptor-ligand pair the successful rupture events were sorted based on the magnitude of the loading rate and ensemble-averaged every 50 rupture events. For each set of binned data the average rupture force and loading rate was calculated and fit to the Bell model [19]. Using a least-squares fit to the linear region of a graph of rupture force against the logarithm of the loading rate the Bell model parameters including the unstressed off-rate $k_{\text{off}}^{{}^{\circ}}$ and the reactive compliance *x_β_* were tabulated [19].

### Western blot analysis

Immunopurified MUC16 or PODXL were separated using a 3–8% Tris-acetate SDS-PAGE gel (Bio-Rad, Hercules, CA) under reducing conditions. Proteins were transferred to an Immuno-blot PVDF membrane and blocked for 30 min using StartingBlock blocking buffer (Thermo Scientific, Waltham, MA). Membranes were stained with either anti-PODXL mAb (3D3) or anti-MUC16 mAb (M11) and rinsed with TBS/0.1% Tween 20, before being incubated with the appropriate horseradish peroxidase (HRP)-conjugated secondary antibody. SuperSignal West Pico chemiluminescent substrate (Life Technologies) was used to develop the immunoblots [11, 12].

### Preparation of MUC16 and PODXL coated microspheres

10 μm polystyrene microspheres (2.5 × 10^7^ microspheres/ml; Polysciences, Warrington, PA, USA) were incubated for overnight at 4°C (constant rotation) with either PODXL or MUC16 immunopurified from SW1990 cell lysate, diluted to a predetermined concentration with binding buffer (0.2M carbonate/bicarbonate buffer 9.2 pH) as described previously [23–25, 48]. Protein coated microspheres were washed 2X with D-PBS and blocked with D-PBS/1% BSA for 60 min at room temperature. Microspheres were re-suspended to a concentration of 2 × 10^6^ microspheres/ml in D-PBS/0.1% BSA for use in flow-based assays. MUC16 and PODXL expression on the surface of microspheres was determined by flow cytometry (Becton, Dickinson, Franklin Lakes, NJ) [23–25, 48].

### Quantification of MUC16 and PODXL site densities on microspheres and SW1990 cells

The site densities of MUC16 and PODXL on microspheres and SW1990 cells were determined by quantitative flow cytometry using a primary anti-MUC16 or anti-PODXL mAb along with an appropriate FITC-conjugated secondary antibody. Background levels were determined by incubating microspheres or cells with properly matched isotype controls [11]. To correlate fluorescent intensity values with Molecules of Equivalent Soluble Fluorochrome (MESF) units, we used the Quantum FITC-5 MESF Kit (Bangs Laboratories, Fisher, IN) and established a calibration curve according to the manufacturer's instructions. The MESF values of MUC16- or PODXL-bearing microspheres or cells were determined using calibration curve. Next, Simply Cellular anti-mouse IgG microspheres (Bangs Laboratories, Fisher, IN) with a known antibody binding capacity (ABC) were utilized to determine the fluorescence to protein (F/P) ratio of the antibody staining process. The F/P ratios for PODXL and MUC16 staining were 2.4 and 1.7 FITC to IgG, respectively. The site densities of MUC16 and PODXL on the microsphere or cell surface were determined by multiplying the net MESF values (anti-MUC16 or anti-PODXL mAb minus the matched isotype control) of MUC16 and PODXL by the inverse of their respective F/P ratios and dividing by the surface area. The surface area of microspheres (10 μm in diameter) is 314 μm^2^. The surface area of SW1990 was estimated to be 1940 μm^2^ as determined by tracing suspended circular cells and using image J for analysis. Flow cytometry assays were performed using a BD FACSCalibur, and data analysis was performed using BD CellQuest Pro software (BD Biosciences, San Jose, CA).

### Selectin patterning via photoligography

A pre-cleaned glass slide was spin coated with micropostit photoresist (S1805, Rohm and Haas, Philadelphia, PA) and exposed to UV through a patterned chrome mask using a previously developed method [26]. To render the patterned slide hydrophobic it was sonicated in 0.1% (v/v) octadecyltrichlorosilane (OTS) for 30 min. The slide was then incubated in FITC-conjugated goat anti-human IgG Fc fragment specific antibody (Sigma, St. Louis, MO) before being immobilized with either recombinant E- or L-selectin Fc chimera at concentrations of 5, 10 and 20 μg/ml. As the goat anti-human IgG Fc antibody recognizes and binds the Fc epitope of chimeric selectins, it was selected to ensure the correct orientation of the E-/L-selectin molecules [26, 42]. The photoresist was removed using 5% photoresist remover (Rohm and Haas) in acetone before the slide was blocked in 1% BSA in D-PBS for at least 1 h prior to the running of experiments. For rolling velocity experiments, the pre-cleaned glass slide was not patterned.

### Quantification of E- and L-selectin site densities

E- and L-selectin site densities were determined using a modified Dissociation-Enhanced Lanthanide Fluorescent Immunoassay (DELFIA) method [26]. First, E- and L-selectin were labeled with europium (Eu^+3^) using the DELFIA Eu-Labelling Kit (PerkinElmer, Waltham, MA) according to the manufacturer's instructions. Labeled proteins were isolated using gel filtration chromatography through a column packed with Sephadex G50 (1 × 10 cm) packed on top of Sepharose 6B (1 × 9 cm) (both from Sigma). 50 mM Tris-HCl (pH 7.8) supplemented with 0.9% sodium chloride and 0.05% sodium azide served as the elution buffer. 1 ml elution fractions were collected from the columns and assayed for protein content using the BCA assay (Thermo Fisher Scientific, Rockford, IL) and Eu^+3^-chelate fluorescence (Supplementary Figure S3A, S3B). Fractions testing positive for both protein and Eu^+3^ were pooled and concentrated by centrifugal filtration through 30,000 MWCO centrifugal filter units (EMD Millipore, Billerica, MA). Concentrated proteins were resuspended in DI water. The final protein concentration was measured using the BCA assay via NanoDrop (Thermo Fisher).

Eu3+ conjugated E- or L-selectin was patterned on glass slides using the identical methodology as was used in the flow-based adhesion or rolling assays. Site densities of E- and L-selectin immobilized on glass surface were assessed by the DELFIA as previously described [26].

### Microfluidic flow based adhesion device fabrication

The microfluidic device was fabricated using standard practices using a previously established methodology [26]. The dimensions of the device channel used in this experiment were 2 cm × 500 μm × 20 μm (length × width × height).

### Microfluidic flow-based adhesion and rolling assays

The microfluidic device was cleaned in ethanol and dried with air before assembly on top of the patterned glass slide. Via a syringe, 0.1% BSA in PBS was introduced into the channel and allowed to flow for 3 min until flow equilibrated. The device was placed on a microscope stage and connected with a syringe pump to control flow. The patches were identified via fluorescence visualization using a Nikon TE300 (Tokyo, Japan). 50 μl of coated microspheres suspended in 0.1% BSA in PBS (2 × 10^6^ microspheres/ml) were added to the inlet channel. The percent binding was determined by dividing the number of interacting microspheres by the total number that passed over each patch. A 20x field of view was used and each perfusion lasted 3 min. For rolling experiments, the microchannel device was assembled to a non-patterned selectin coated glass slide, with all other conditions remaining the same. Rolling velocities were determined by dividing the distance the microsphere center of mass traveled by the time taken to travel that distance for each condition tested [19, 25].

### Mathematical model of 2-D selectin-mediated cell adhesion on micropatches

The model for selectin binding to 2-D micropatches utilized here was discussed in detail previously [26]. All calculations are similar with the exception of *k_off_*, which was calculated based on the one-pathway model for step-wise bond rupture [33]. The step-wise model was chosen as binding was found to be mediated by a larger number bonds (>5) and simultaneous bond rupture was unlikely. The kinetic constants used were determined via single molecule force spectroscopy.

### Statistical analysis

Data are expressed as means ± S.E.M. or S.D. of at least 3 independent experiments. Statistical significance of differences between means was determined by Student's *t* test or one-way analysis of variance followed by the either the Tukey or Sidak test for multiple comparisons, where appropriate.

## SUPPLEMENTARY FIGURES AND TABLES


