# Supplementary material for: Distinct kinetic and mechanical properties govern mucin 16- and podocalyxin-mediated tumor cell adhesion to E- and L-selectin in shear flow
Source: Oncotarget. 2015 Jul 28;6(28):24842–55. doi: 10.18632/oncotarget.4704 (PMC4694797; doi:10.18632/oncotarget.4704)
Supplement: Supplementary file 1 [file oncotarget-06-24842-s001.pdf]

SUPPLEMENTARY FIGURES AND TABLES

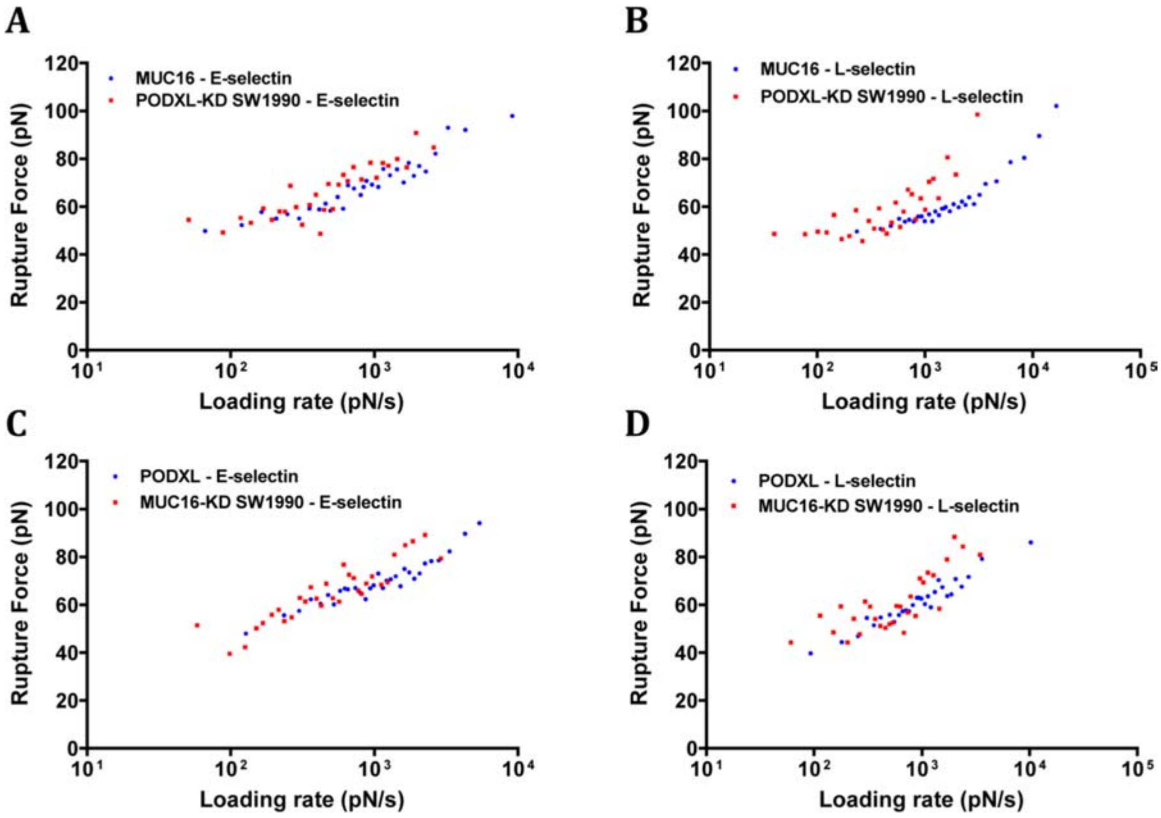

Supplementary Figure S1: Micromechanical properties of MUC16 and PODXL binding to E-/L-selectin for both protein-protein and cell-protein systems. Rupture force as a function of loading rate was measured for the interaction between E- or L-selectins and immunopurified MUC16 or PODXL as well as MUC16-expressing (PODXL-KD) or PODXL-expressing (MUC16-KD) SW1990 cells. Data represent the mean ± S.E.M. of 3–4 independent experiments for each binding pair.

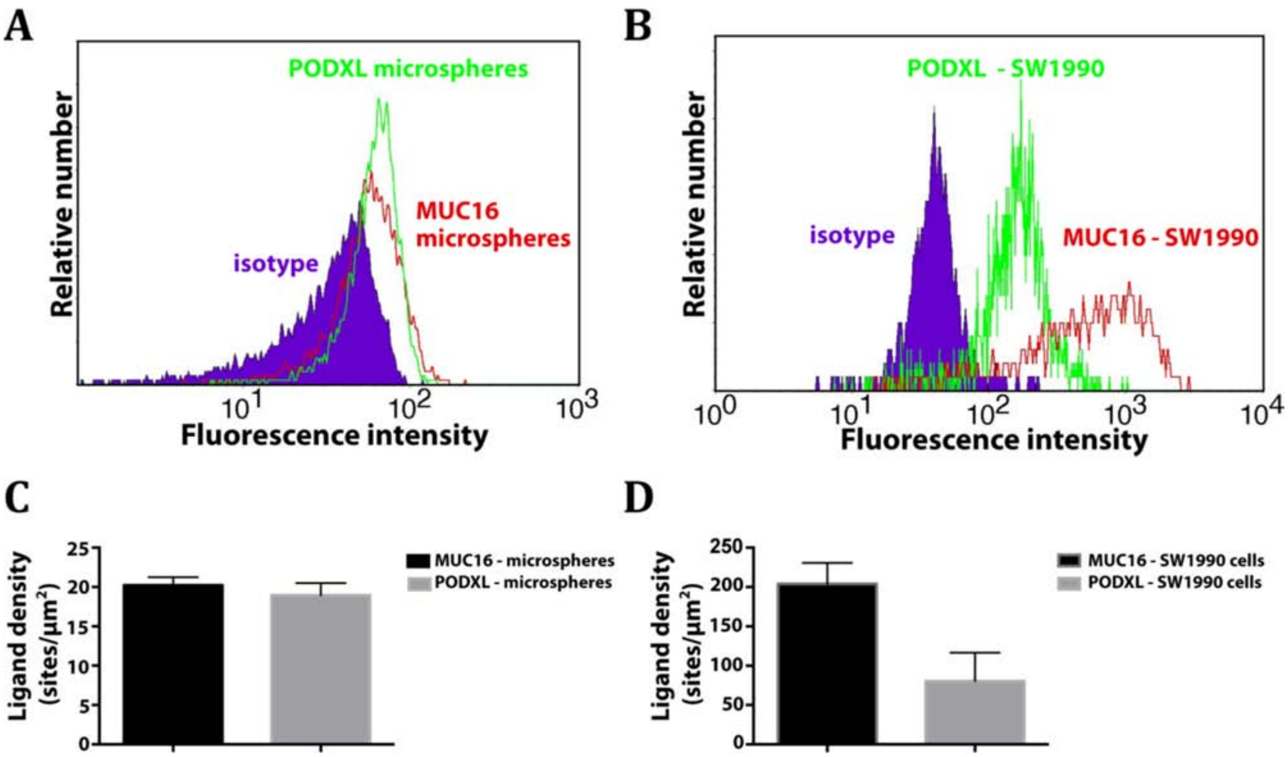

Supplementary Figure S2: PODXL and MUC16 site density on microspheres and SW1990 cells as determined by quantitative flow cytometry. Representative flow cytometric histograms of MUC16 and PODXL on **A.** protein-coated microspheres and **B**. SW1990 pancreatic cancer cells. Microspheres and SW1990 cells were stained by indirect single-color immunofluorescence using anti-PODXL or anti-MUC16 mAbs or their respective isotype control antibodies (purple). Using the Quantum FITC-5 MESF Kit in conjunction with Simply Cellular anti-mouse IgG microspheres (Bangs Laboratories, Fisher, IN), the site density of MUC16 and PODXL on **C.** protein-coated microspheres and **D**. SW1990 cells were calculated. Data represent the mean ± S.E.M. of at least four independent experiments.

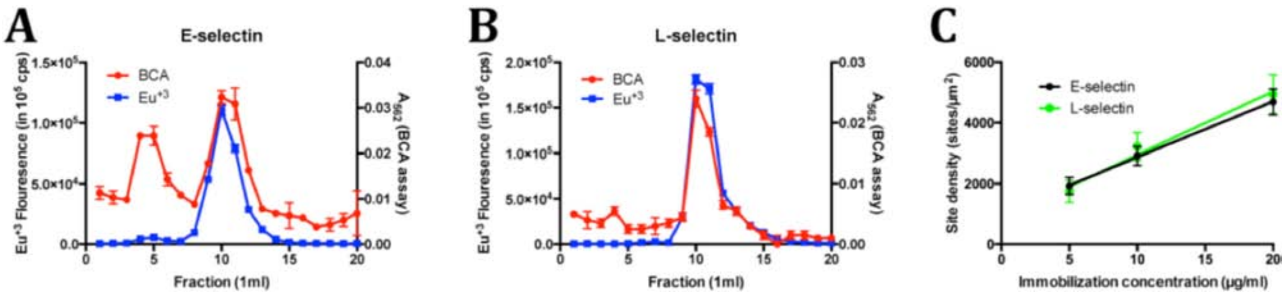

Supplementary Figure S3: Determination of E- and L- selectin surface site density. **A.** E-selectin or **B.** L-selectin was conjugated with europium (Eu^{+3}) using the DELFIA Eu-Labelling Kit and purified via gel filtration chromatography. Elution fractions positive for both protein (absorbance at 562 nm following BCA assay) and Eu^{+3} content (high time-resolved fluorescence in RFU) were pooled and concentrated. **C.** The purified europium-conjugated E- or L-selectin was used to determine the surface site density. Data represent the mean ± S.E.M. of at least four independent experiments.

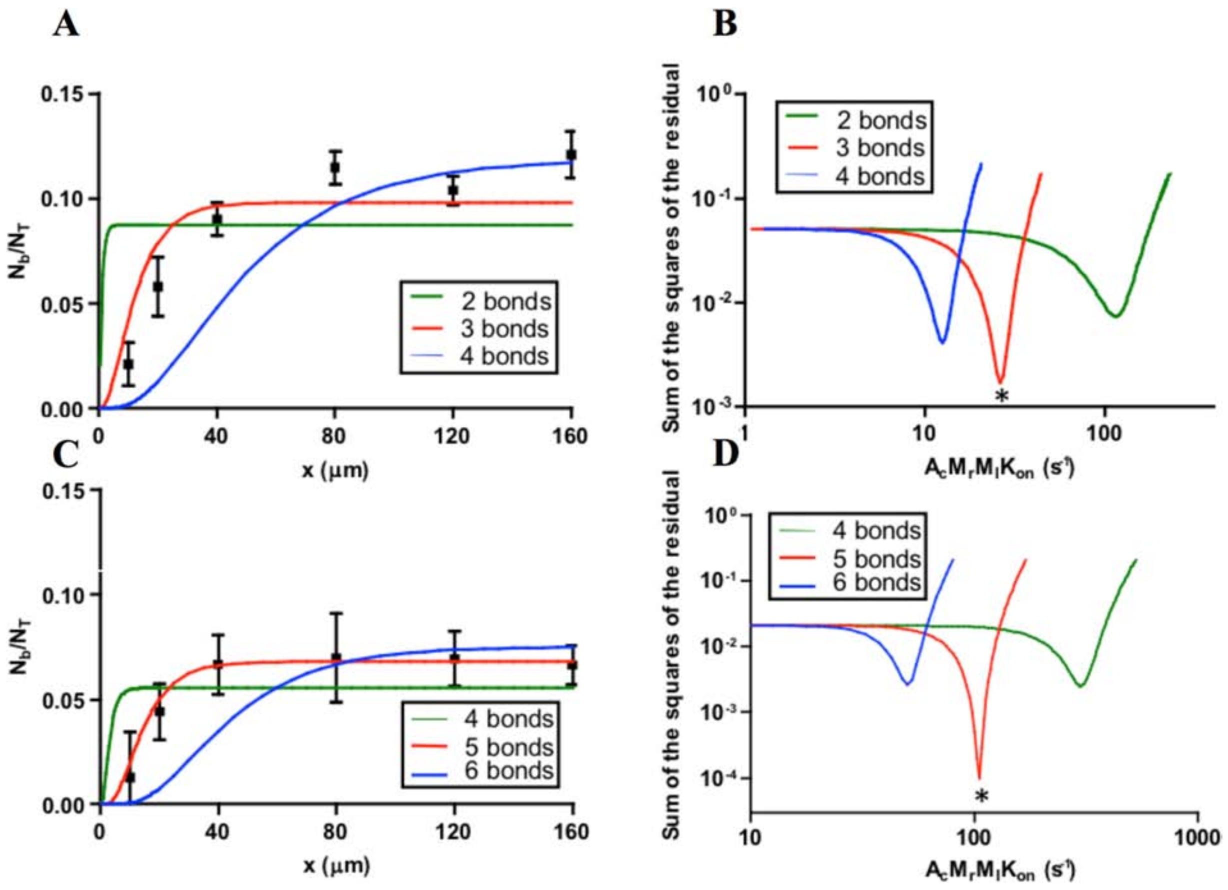

Supplementary Figure S4: The multi-bond model determines the number of bonds and lumped affinity ($A_cM_rM_lK_{on}$) needed to mediate PODXL-coated microsphere tethering on E-selectin in shear flow. Fitting of the multi-bond model to experimental data for PODXL-coated microspheres interacting with 3000 sites/µm² E-selectin at 0.5 dyn/cm² **A.** and at 1 dyn/cm² **C.** The sum of squares of residual **B** and **D.** was calculated by fitting the model to the experimental data shown in (**A**) and (**C**), respectively. The lumped binding affinity ($A_cm_rm_lk_{on}$) for the optimal fit is marked by (*) on (**B**) and (**D**).

Supplementary Table S1. $A_cM_rM_lK_{on}$ for MUC16- and PODXL- coated microspheres tethering on E-/L-selectin coated surface as predicted by the multi-bond model

Binding pair	$A_cM_rM_lK_{on}$ (s^{-1})		
	Shear stress (dyn/cm^2)		
	0.5	1	2
MUC16-E-selectin	10.1	42.6	151.7
MUC16-L-selectin	16.1	47.1	124.8
PODXL-E-selectin	26.3	105.1	300.2
PODXL-L-selectin	8.6	38.1	115.7

The multi-bond model was fitted to the experimental data and optimized for both the number of bonds needed to achieve tethering and lumped affinity ($A_cM_rM_lK_{on}$) for MUC16- and PODXL-coated microspheres tethering to 3000 sites/µm^2 E- or L-selectin coated patches.

Supplementary Table S2. Comparison of the binding parameters for ligand - E/L-selectin pairs

Binding pair	$k^{\circ}_{off}(s^{-1})$	x_{β}(nm)	Rupture force @1000 pN/s (pN)
MUC16–E-selectin	0.26 ± 0.08	0.34 ± 0.02	71
PODXL–E-selectin	0.33 ± 0.05	0.33 ± 0.01	69
PMN (PSGL-1)–E-selectin*	0.31 ± 0.06	0.12 ± 0.01	156
sLeX–E-selectin§	0.30	0.5	48
MUC16–L-selectin	0.82 ± 0.04	0.36 ± 0.01	56
PODXL–L-selectin	0.49 ± 0.04	0.35 ± 0.02	62
PMN (PSGL-1)–L-selectin*	0.85 ± 0.10	0.17 ± 0.01	93
sLeX–L-selectin§	6	0.45	29

*Data presented from Hanley *et al*. [42] with Bell model kinetic constants for linear region (100–10,000 pN/s) and rupture forces estimated from provided figures.
§Data presented from Zhang *et al*. [43] with Bell model kinetic constants for linear region (100–10,000 pN/s) and rupture forces estimated from provided figures. Where appropriate, data shown as the mean ± S.D.
